# Quantitative Analysis of HER2 Amplification by Droplet Digital PCR in the Follow-Up of Gastric Cancer Patients Being Treated with Trastuzumab after Surgery

**DOI:** 10.1155/2019/1750329

**Published:** 2019-01-31

**Authors:** Yanzhuo Liu, Maozhu Yang, Tao Jiang, Chunbin Lan, Hao Yuan, Guiquan Li, Guiqing Jia, Kang Wang, Gaoping Zhao

**Affiliations:** ^1^Department of Gastrointestinal Surgery, Sichuan Academy of Medical Sciences & Sichuan Provincial People's Hospital, School of Medicine, University of Electronic Science and Technology of China, Chengdu 610000, China; ^2^Institute of Chengdu Biology and Sichuan Translational Medicine Hospital, Chinese Academy of Sciences, Chengdu 610000, China; ^3^Department of General Surgery, Sichuan Academy of Medical Sciences & Sichuan Provincial People's Hospital, School of Medicine, University of Electronic Science and Technology of China, Chengdu 610000, China; ^4^Department of General Surgery, Qionglai Medical Center Hospital, Chengdu, Sichuan Province 610000, China

## Abstract

**Background:**

Circulating tumor DNA (ctDNA) derived from tumors is a promising biomarker for monitoring tumor status and evaluating therapeutic effects and prognosis. We studied the plasma human epidermal growth factor receptor 2 (HER2) amplification in gastric cancer (GC) patients by droplet digital PCR (ddPCR) during therapy with trastuzumab.

**Methods:**

A total of 12 patients were recruited after surgery. All patients received FOLFOX chemotherapy combined with trastuzumab as a treatment regimen. During the 12 months of the follow-up period, using elongation factor Tu GTP binding domain containing 2 (EFTUD2) as a reference gene, plasma HER2 to EFTUD2 ratios (the HER2 ratio) were determined for each patient every 2 months by ddPCR.

**Results:**

The concordance rate of HER2 amplification examined in plasma and formalin-fixed paraffin-embedded (FFPE) samples with ddPCR was 81.4%, with a sensitivity of 76.5% and a specificity of 83.8%. Plasma HER2 ratios were correlated with the primary tumor size (*p* < 0.01). A significant decrease in the plasma HER2 ratio was found after two months of treatment (*p* < 0.0001). Nine patients experienced partial response, and three patients had stable disease. Seven patients had progressive disease (PD) during follow-up, and four of them had died. The median progression-free survival (PFS) was 9.8 months. For each patient who developed PD, the plasma HER2 ratio was approximately 2.3-4.1 times higher than the cut-off value at the time of PD, which was the highest during the whole follow-up period.

**Conclusion:**

Longitudinal monitoring for the plasma HER2 ratio by ddPCR in the clinical courses of GC patients holds great promise for use as an indicator of tumor progression and treatment efficacy.

## 1. Introduction

According to the latest cancer statistics, gastric cancer (GC) is the second most common cancer in terms of incidence and mortality in China [1]. Except for the surgery and radiotherapy, a combination of systemic chemotherapy and platinum-containing regimens represents the gold standard of first-line treatment for advanced GC [2–4], which could significantly improve the overall survival (OS) and quality of life. However, its potential benefit has reached a plateau. Recently, accumulating evidences regarding the efficacy of novel targeted therapies in the treatment of GC have been reported [5–9], such as human epidermal growth factor receptor 2 (HER2), epidermal growth factor receptor (EGFR), and vascular endothelial factor and its receptor (VEGF/VEGFR) based on the targets. Although the results were mostly not promising, two meta-analyses of randomized trials revealed that HER2 was the most effective of targeted drugs for its outstanding improvement of OS and progression-free survival (PFS) [10, 11].

To date, trastuzumab has been recommended by the European Medicines Agency and the United States Food and Drug Administration (FDA) as a new treatment option for patients with HER2-positive GC [12]. It was reported that HER2 overexpression occurs in 7–42% of tumor tissues in GC patients [13]. The detection of HER2 was routinely performed in resected tumors using immunohistochemistry (IHC) and fluorescence in situ hybridization (FISH). However, the distribution of HER2-expressing cells is known to be heterogeneous in GC [14]. And a regular monitoring of the HER2 status to evaluate the prognosis and reconsider treatment strategies was potentially required, but clinical monitoring of molecular mutations or changes in expression through repeated biopsy of tumor tissues would be difficult, because of its invasive nature, high incidence of inducing tumor metastasize, and so on. Hence, it is critical to find alternative methods to determine the issue, by using other nonbiopsy specimens which can be acquired easily and dynamically, such as plasma, serum, or urine.

Circulating tumor DNA (ctDNA) derived from tumors and released into the bloodstream is a promising biomarker for cancer screening and prognosis and for monitoring the status of tumors and the efficacy of anticancer therapies [15–17]. These molecular alterations could be analyzed by various methods, including quantitative real-time PCR (Q-PCR), next-generation sequencing (NGS), or droplet digital PCR (ddPCR). Among these techniques, ddPCR was proven to be more precise, sensitive, and less affected by PCR inhibitors and did not need internal/external normalization when compared with others [18, 19]. Some studies have demonstrated the superior accuracy of ddPCR for measuring copy number variation (CNV) [20, 21]. And it has also been proven that the detection of HER2 in ctDNA in GC by ddPCR could precisely estimate the HER2 status of tumors [22, 23].

In this study, we recruited GC patients who received treatment with FOLFOX chemotherapy (oxaliplatin (L-OHP)+calcium folinate (CF)+5-fluorouracil (5-Fu)) and trastuzumab and detected the HER2 copy number status by ddPCR in plasma samples every 2 months during the 12 months of follow-up, to evaluate its potential clinical use as a biomarker for monitoring the tumor status and treatment responses during the treatment period.

## 2. Materials and Methods

### 2.1. Patients and Blood Samples

**Figure 1:**
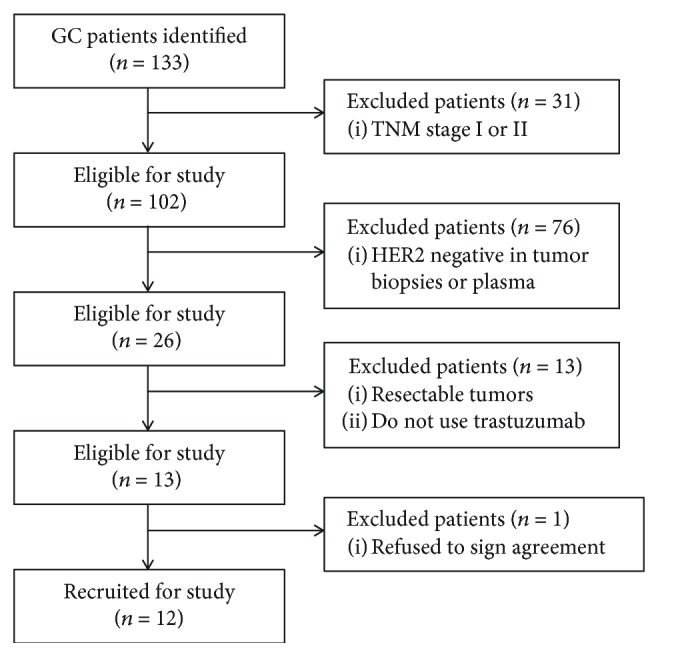
Enrollment of gastric cancer patients. Flow chart of patients in the study. GC: gastric cancer; HER2: human epidermal growth factor receptor 2.

A total of 133 patients diagnosed with gastric cancer (GC) from Sichuan Provincial People's Hospital between May 2015 and January 2016 were considered for recruitment in the present study. Enrollment criteria included the following: (1) patients were categorized as stage III or IV according to TNM stage classification, (2) patients received treatment with trastuzumab, (3) patients' tumor biopsies and blood samples were HER2 positive as detected by ddPCR, and (4) by a signed agreement, patients agreed to provide additional blood samples and received clinical examination including computed tomography (CT) imaging every 2 months during the 12 months of follow-up. Finally, there were 111 patients excluded as described in Figure 1. The present study was approved by the ethics committee of Sichuan Provincial People's Hospital. All patients provided a written informed consent form to approve the use of their blood samples and clinical data for research purposes.

For plasma, 10 mL peripheral blood was collected in Streck Cell-Free DNA BCT tubes (Streck, Omaha, NE), which enabled the stability of circulating DNA in whole blood for at least 7 days at room temperature [24]. Within 8 hours, plasma was separated by centrifugation at 2000 × g for 10 minutes; the supernatant was followed by a second centrifugation at 12000 × g for 20 minutes. Then, the plasma was stored at −80°C until use.

### 2.2. DNA Extraction

Circulating cell-free DNA (cfDNA) was isolated from 4 mL of plasma using the QIAamp Circulating Nucleic Acid Kit (Qiagen, Germany) according to the manufacturer's instructions. cfDNA was eluted from spin columns in 40 *μ*L elution buffer as provided with the kit; the concentration was approximately 10-30 ng/*μ*L. And then cfDNA was used in ddPCR analysis without storage at −80°C.

### 2.3. ddPCR Workflow

For each ddPCR assay, 5 *μ*L cfDNA sample, 10 *μ*L 2× ddPCR supermix for probes (Bio-Rad), 1 *μ*L primer/probe mix, and 4 *μ*L RNase-free water were added in a 20 *μ*L reaction mixture. The primer/probe mix for HER2 and elongation factor Tu GTP binding domain containing 2 (EFTUD2) were designed based on Kinugasa et al. [22] and custom made by Thermo Fisher Scientific Inc. For HER2 assay, primer sequences are as follows: forward, 5′-ACAACCAAGTGAGGCAGGTC-3′, and reverse, 5′-GTATTGTTCAGCGGGTCTCC-3′; probe sequences are as follows: 5′-FAM-CCCAGCTCTTTGAGGACAAC-MGB-NFQ-3′. For the EFTUD2 assay, primer sequences are as follows: forward, 5′-GGTCTTGCCAGACACCAAAG-3′, and reverse, 5′-TGAGAGGACACACGCAAAAC-3′; probe sequences are as follows: 5′-VIC-GGACATCCTTTGGCTTTTGA-MGB-NFQ-3′. Then, the 20 *μ*L mixture and 70 *μ*L droplet generation oil for probes (Bio-Rad) were respectively loaded into the sample wells and oil wells of a disposable droplet generator cartridge (Bio-Rad). After that, droplets were generated by a QX200 droplet generator device (Bio-Rad) and carefully transferred to a 96-well PCR plate (Eppendorf). The cycling conditions were 95°C for 10 minutes, 40 cycles of 95°C for 15 seconds and 58°C for 1 minute, and a final step at 98°C for 10 minutes. At the end of the PCR reaction, droplets were read in the QX200 droplet reader and analyzed using the QuantaSoft™ version 1.7.4 software (Bio-Rad). In addition, a no template control (NTC) was included in every assay.

### 2.4. Statistical Analysis

The statistical analyses were performed using the SPSS version 19.0 software. The Mann-Whitney *U* test was used to compare significant differences in HER2 ratios of different blood samples. Continuous data are presented as means ± standard deviation. Correlation coefficients were calculated using the Spearman or Pearson method. A *p* value of less than 0.05 was considered to be significant.

## 3. Results

### 3.1. Patient Characteristics

A total of 12 patients with advanced GC were enrolled in the present study, comprised of 7 males and 5 females. All patients agreed to receive the treatment regimen of FOLFOX chemotherapy combined with trastuzumab. All patients were categorized as TNM stage IV and had small or extensive lymph node metastasis, eight patients had liver metastasis, and four patients had peritoneum seeding metastasis; the pathologic differentiation grade was either moderate or poor (Table 1). The median age was 63.4 years (range, 36-79 years). The tumor location of GC includes the antrum, cardia, and body. The median tumor size was 4.6 cm (range, 4-6.5 cm) (Table 1). For each patient, GC paraffin-embedded tissue samples were obtained after surgical resection, and one blood sample was collected 3 days before surgery. Additional blood samples were collected as per the signed agreement, unless the patient died during follow-up. At the same time, CT imaging was performed to evaluate treatment responses according to the Response Evaluation Criteria in Solid Tumors (RECIST), version 1.1 [25]. The details regarding progressive disease (PD) and subsequent management were recorded. Moreover, the clinicopathological classification and staging were determined according to the World Health Organization pathological classification of tumors. By the end of the last follow-up, seven patients experienced progressive disease and four patients had died. The median progression-free survival (PFS) was 9.8 months (Table 2).

### 3.2. Technical Sensitivity and Specificity of HER2 Ratio Assays in the cfDNA

According to a previous study, EFTUD2, which had a highly stable copy number ratio with the ERBB2 locus and located in the same region with HER2 on chromosome 17, was used as a reference for the ddPCR assay [26]. A cut-off value for plasma and tissue HER2 to EFTUD2 ratios (the HER2 ratio) was set at 1.2 based on studies by Kinugasa et al. and Gevensleben et al. [22, 26].

The HER2 status in formalin-fixed paraffin-embedded (FFPE) samples was determined by routine IHC and FISH methods according to the published criteria [27]. The sensitivity and specificity of the ddPCR assays in measuring HER2 ratios in plasma and FFPE samples were determined from 102 GC patients (Figure 1). When the droplet number is less than 10000, the data would be considered invalid and a repeat detection would be performed. Overall, 39 and 63 patients were HER2-postive and HER2-negative, respectively, by IHC/FISH. Similar results were obtained by ddPCR; 37 patients were determined as HER2-postive when a HER2 ratio was higher than the cut-off value (1.2). The sensitivity and specificity were 87.1% (34/39) and 95.2% (60/63), respectively (Table 3). The concordance rate was 92.2% (94/102). Compared with tumor tissue samples, the sensitivity and specificity of ddPCR for HER2 status in plasma were 76.5% (26/34) and 83.8% (57/68), respectively (Table 3). The concordance rate of HER2 amplification examined in plasma and FFPE samples with ddPCR was 81.4% (83/102). These results demonstrated that analysis of cfDNA with ddPCR has good accuracy screening for the acquisition of HER2 amplification in GC cancer.

### 3.3. Association with Tumor Characteristics

**Figure 2:**
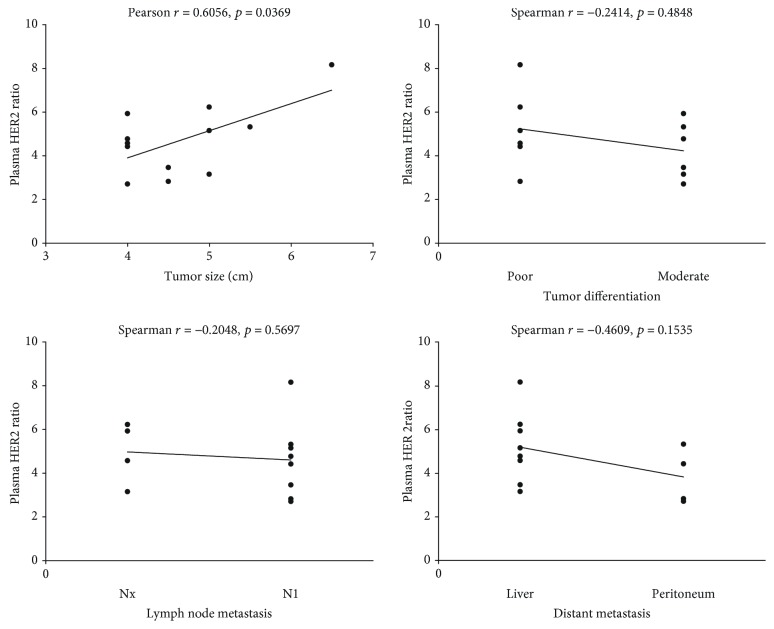
Relationship between tumor characteristics and the plasma HER2 ratio in patients with GC. The correlation analysis was performed between the plasma HER2 ratio before receiving treatment and tumor characteristics including tumor size, differentiation, lymph node metastasis, and distant metastasis, using the Spearman or Pearson method. A *p* value of less than 0.05 was considered to be significant.

The HER2 ratio in plasma samples of GC patients before receiving treatment was 4.74 ± 1.60 (range, 2.84-8.19). As shown in Figure 2, the correlation analysis was performed between HER2 status before receiving treatment and some clinicopathological characteristics including tumor size, differentiation, lymph node metastasis, and distant metastasis. After being analyzed separately, only tumor sizes were found to be positively correlated with HER2 ratios (Pearson *r* = 0.6056, *p* = 0.0369). No correlation was found between HER2 ratios before receiving treatment and tumor differentiation, lymph node metastasis (N1 or Nx), or distant metastasis (the liver or peritoneum) (Spearman *r* = −0.2414, *p* = 0.4848; *r* = −0.2048, *p* = 0.5697; and *r* = −0.4609, *p* = 0.1535, respectively). Moreover, a significant decrease in the HER2 ratio (1.81 ± 0.57, range, 1.18-2.91) was found in the plasma of GC patients after 2 months of FOLFOX chemotherapy combined with trastuzumab (*p* < 0.0001). These results suggested that HER2 ratios may be used as additional indicators for evaluating the effect of tumor therapy or tumor size in patients with HER2-positive GC.

### 3.4. Longitudinal Monitoring for HER2 Status in the Plasma of GC Patients Treated with Trastuzumab

To investigate whether HER2 ratios in cfDNA can be used to monitor the treatment response or tumor progression longitudinally, plasma samples were analyzed at specified time points during the course of their treatment, together with CT imaging. During the 12 months of follow-up, three patients had a best response of stable disease and 9 patients had a partial response (PR) (Table 2). Seven patients were found to have progressive disease at 6 months, 8 months, or 10 months posttreatment. Four of them had died before 10 months or 12 months during follow-up.

**Figure 3:**
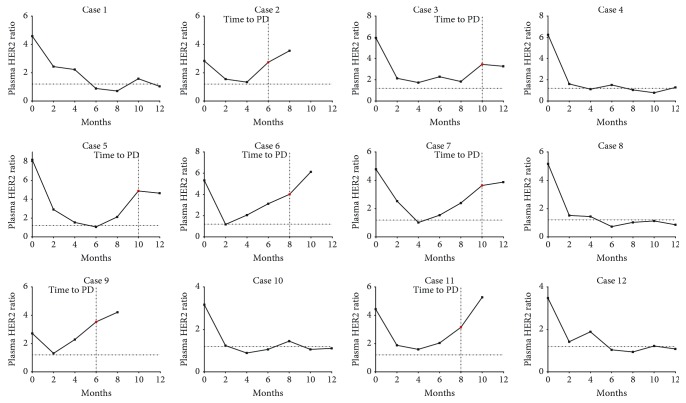
Monitoring plasma HER2 ratios during clinical courses of GC patients. Changes in plasma HER2 ratios during the 12 months of the follow-up period. Seven patients (cases 2, 3, 5, 6, 7, 9, and 11) were found to have progressive disease (PD), and four patients (cases 2, 6, 9, and 11) had died. The increases in their plasma HER2 ratios were observed at the time of PD, which was approximately 2.3-4.1 times higher than the cut-off value. The 0 month means presurgery. The horizontal dotted line represents the cut-off value of HER2 ratios (*n* = 1.2).

For patients who had a best response of SD or PR, the HER2 ratio in plasma before receiving treatment was 3.01 ± 0.41 (range, 2.72-3.48) and 5.32 ± 1.40 (range, 3.17-8.19), respectively. Significant difference was found between these groups (*p* = 0.0182). Moreover, after 2 months of treatment, the decrease in the HER2 ratio in patients who had a PR was more significant (3.38 ± 1.20; range, 1.93-5.28) when compared with patients who had a SD (1.58 ± 0.41; range, 1.29-2.06). The median survival time for patients who had a SD or PR was 8 months and >11.6 months, respectively. These results suggested that a high level of HER2 gene amplification was associated with better response and longer survival time in HER2-positive advanced GC treated with trastuzumab. Seven patients showed a high plasma HER2 ratio at the time of progressive disease during the follow-up period, which was approximately 2.3-4.1 times higher than the cut-off value (Table 2 and Figure 3). It was supposed that these might be induced by the proliferation of tumor cells with HER2 amplification or acquisition of HER2 amplification in tumor cells. Furthermore, except for the case 2 and case 3, five cases (case 5, case 6, case 7, case 9, and case 11) showed an obvious increasing trend of plasma HER2 ratio at least 2 months before the time of tumor progression, which was approximately 1.7-2 times higher than the cut-off value (Table 2 and Figure 3). In comparison with that, five patients (case 1, case 4, case 8, case 10, and case 12) did not develop progressive disease; a remarkable and continuous decrease in the plasma HER2 ratio was found after prompt treatment with FOLFOX chemotherapy plus trastuzumab during follow-up (Table 2 and Figure 3). Taken together, these results suggest that trastuzumab is an effective reagent for the treatment of HER2-positive GC, and the plasma HER2 ratio determined by ddPCR may serve as a biomarker for tumor progression in GC patients who received treatment with trastuzumab.

## 4. Discussion

In the past few years, the activity of several driver mutations has been demonstrated in approximately 40% of GC patients [27, 28]. A number of randomized phase III trials testing target therapies in the treatment of advanced GC patients have been performed in clinics, including anti-HER2 targeted agents, angiogenesis inhibitors, EGFR inhibitors, and mTOR inhibitors [5–7, 9]. However, only the use of trastuzumab had shown significant benefits in HER2-positive tumors. To date, trastuzumab is the only targeted agent to have obtained US FDA and European Medicines Agency (EMA) approval for the treatment of HER2-overexpressing advanced GC [12]. As expected, in the present study, through a regular follow-up and treatment with FOLFOX chemotherapy plus trastuzumab, most patients (9/12) achieved a PR, and the median progress-free survival was 9.8 months at the last follow-up.

HER2 positivity in GC widely ranges from 7% to 42%, because of the heterogeneity in HER2 amplification in tumors [13]. A previous study has demonstrated that the level of HER2 gene amplification could predict the outcome in GC patients treated with trastuzumab; the optimal cut-off value of the HER2/CEP17 ratio in tumor tissues for predicting survival longer than 12 months was 4.45 [29]. In the present study, we also found that GC patients who had a HER2/EFTUD2 ratio > 3 in plasma were more likely to have a better response and survive longer during follow-up. However, it is also important to monitor the expression status of HER2 for making a proper adjustment of treatment strategies, since the HER2 expressions in tumors are frequently changed during the treatment in the clinic because of genetic differentiation [14, 30]. The routinely used methods of IHC/FISH are not suitable for monitoring HER2 status during the clinical course, because it is difficult to regularly obtain biopsy samples. In that case, ctDNA in the blood was considered to hold great promise for solving this issue. Indeed, a number of studies have proven that the presence of certain ctDNA markers could be used as an indicator and predictor of tumor progression or drug resistance, in non-small-cell lung cancer, colon cancer, breast cancer, and gastric cancer [30, 31].

The ddPCR technique was considered to be or would be the gold standard in the application of liquid biopsy, because it has shown superior precision and sensitivity while detecting low concentration of target nucleic acid molecules [18, 19]. Kinugasa et al. have made a first step to use ddPCR to evaluate HER2 amplification in serum of GC patients [22]. In the present study, we identified that 37 out of 133 patients (27.8%) were HER2-positive by IHC/FISH. And our results have shown outstanding sensitivity and specificity, with a concordance rate of 81.4% for determining HER2 status by ddPCR between FFPE and plasma samples, which was higher than a concordance rate of 62.5% reported by Kinugasa et al. [22]. We suppose that the use of Streck Cell-Free DNA BCT tubes for sample collection and bigger sample volume for ctDNA extraction leads to these different results. Since the concentration of ctDNA in plasma is low and the half-life of cfDNA is less than 1.5 h, the use of common EDTA tubes and insufficient sample volume might confound the results. Thus, our results suggest that the dynamic monitoring of HER2 amplification by ddPCR is technically feasible, and it might become a standard method for analyzing ctDNA in GC.

A previous study reported by Shoda et al. has explored the effectiveness of monitoring HER2 amplification in plasma of GC patients by ddPCR. It demonstrated that the plasma HER2 status might be a useful marker for monitoring tumor status. However, most patients did not have blood samples after surgery, and only 6 cases have repeated measurements of the HER2 ratios in plasma which are collected discontinuously during the follow-up period [23]. In the present study, we had a regular blood collection and medical examination for all patients every 2 months during the treatment with FOLFOX chemotherapy plus trastuzumab. Our results showed that plasma HER2 ratios were significantly decreased after treatment in most patients. During the 12 months of the follow-up period, a significant increase in plasma HER2 ratios was found to be associated with tumor progression. Similar to the study by Shoda et al., it also could suggest that the monitoring of HER2 status by ddPCR could offer an insight to predict tumor progression and evaluate treatment efficacy in the clinical courses of GC patients.

Although the present study is a prospective study, it has quite a few limitations. First of all, the number of recruited patients was small and had little or no heterogeneity in TNM stage, tumor size, differentiation, lymph node, and distant metastasis. Secondly, it was an observational study without a proper control group, and we did not give additional treatment or adjust medications according to the frequently changed HER2 status in plasma. Thus, we still do not even know how to make a proper adjustment and do not have any direct evidence to demonstrate that the proper adjustments of treatment strategies according to HER2 status would have benefits for the outcomes of GC patients. Thirdly, most patients had frequent changes of plasma HER2 status during the follow-up, such as increasing from HER2-negative to HER2-positive or decreasing from HER2-positive to HER2-negative. We could not confirm what factors affected the results, such as the proliferation of HER2-positive tumor cells, the acquisition of HER2 amplification in tumor cells, or the set of cut-off values. And we also do not know whether these results could reflect the real status of tumor progression if without the related biopsy samples. Thus, any final conclusions regarding the effectiveness of monitoring HER2 status by ddPCR in GC patients still could not be drawn. Further studies are required.

## 5. Conclusion

In conclusion, longitudinal monitoring for the plasma HER2 ratio by ddPCR in the clinical courses of GC patients holds great promise for use as an indicator of tumor progression and treatment efficacy.

## Figures and Tables

**Table 1:** Clinicopathological characteristics of all patients.

Patient ID	Age	Gender	Tumor location	Tumor size (cm)	Differentiation	Lymph node metastasis	Distant metastasis	Stage	TNM	Treatment
1	53	Female	Antrum	4	Poor	Positive	Liver	IV	T4aNxM1	FOLFOX+trastuzumab
2	36	Male	Antrum	4.5	Poor	Positive	Peritoneum	IV	T4N1M1	FOLFOX+trastuzumab
3	64	Female	Cardia	4	Moderate	Positive	Liver	IV	T4NxM1	FOLFOX+trastuzumab
4	70	Female	Cardia	5	Poor	Positive	Liver	IV	T4bNxM1	FOLFOX+trastuzumab
5	71	Male	Body	6.5	Poor	Positive	Liver	IV	T4N1M1	FOLFOX+trastuzumab
6	65	Male	Body	5.5	Moderate	Positive	Peritoneum	IV	T4bN1M1	FOLFOX+trastuzumab
7	79	Male	Antrum	4	Moderate	Positive	Liver	IV	T4N1M1	FOLFOX+trastuzumab
8	72	Male	Antrum	5	Poor	Positive	Liver	IV	T4N1M1	FOLFOX+trastuzumab
9	56	Male	Cardia	4	Moderate	Positive	Peritoneum	IV	T4bN1M1	FOLFOX+trastuzumab
10	59	Female	Cardia	5	Moderate	Positive	Liver	IV	T4aNxM1	FOLFOX+trastuzumab
11	64	Female	Body	4	Poor	Positive	Peritoneum	IV	T4N1M1	FOLFOX+trastuzumab
12	72	Male	Antrum	4.5	Moderate	Positive	Liver	IV	T4N1M1	FOLFOX+trastuzumab

FOLFOX = oxaliplatin (L-OHP)+calcium folinate (CF)+5-fluorouracil (5-Fu).

**Table 2:** Dynamics of plasma HER2 ratios in GC patients by ddPCR during the follow-up period.

Patient ID	HER2 ratios							Best response	PFS (months)
	0 months	2 months	4 months	6 months	8 months	10 months	12 months		
1	4.59	2.44	2.23	0.89	0.71	1.58	1.04	PR	12
2	2.84	1.55	1.34	**2.74** ^∗^	3.56	—	—	SD	6
3	5.95	2.14	1.74	2.29	1.84	**3.46** ^∗^	3.28	PR	10
4	6.25	1.61	1.12	1.52	1.05	0.78	1.28	PR	12
5	8.19	2.91	1.54	1.06	2.12	**4.88** ^∗^	4.64	PR	10
6	5.34	1.18	2.06	3.13	**4.02** ^∗^	6.14	—	PR	8
7	4.79	2.53	1.02	1.54	2.4	**3.64** ^∗^	3.88	PR	10
8	5.17	1.52	1.44	0.73	1.03	1.13	0.86	PR	12
9	2.72	1.31	2.29	**3.54** ^∗^	4.22	—	—	SD	6
10	3.17	1.24	0.89	1.06	1.45	1.06	1.11	PR	12
11	4.44	1.88	1.59	2.04	**3.15** ^∗^	5.28	—	PR	8
12	3.48	1.42	1.89	1.04	0.94	1.22	1.08	SD	12

SD: stable disease; PR: partial response; PFS: progress free survival; ∗ means time to progressive disease.

**Table 3:** Concordance of plasma and tumor tissue sample results for HER2 status from GC patients by ddPCR or IHC/FISH.

	HER2 in FFPE with ddPCR		Total	Sensitivity	Specificity
	Positive	Negative			
HER2 in FFPE with IHC/FISH					
Positive	34	5	39	87.2%	95.2%
Negative	3	60	63		
HER2 in ctDNA with ddPCR					
Positive	26	8	34	76.5%	83.8%
Negative	11	57	68		

## Data Availability

The data used to support the findings of this study are available from the corresponding author upon request.

## References

[1] Siegel R., Naishadham D., Jemal A. (2013). Cancer statistics, 2013. *CA: a Cancer Journal for Clinicians*.

[2] Chen W. W., Wang F., Xu R. H. (2013). Platinum-based versus non-platinum-based chemotherapy as first line treatment of inoperable, advanced gastric adenocarcinoma: a meta-analysis. *PLoS One*.

[3] Price T. J., Shapiro J. D., Segelov E. (2012). Management of advanced gastric cancer. *Expert Review of Gastroenterology & Hepatology*.

[4] Koizumi W., Narahara H., Hara T. (2008). S-1 plus cisplatin versus S-1 alone for first-line treatment of advanced gastric cancer (SPIRITS trial): a phase III trial. *The Lancet Oncology*.

[5] Bang Y.-J., van Cutsem E., Feyereislova A. (2010). Trastuzumab in combination with chemotherapy versus chemotherapy alone for treatment of HER2-positive advanced gastric or gastro-oesophageal junction cancer (ToGA): a phase 3, open-label, randomised controlled trial. *The Lancet*.

[6] Ohtsu A., Ajani J. A., Bai Y. X. (2013). Everolimus for previously treated advanced gastric cancer: results of the randomized, double-blind, phase III GRANITE-1 study. *Journal of Clinical Oncology*.

[7] Aprile G., Bonotto M., Ongaro E., Pozzo C., Giuliani F. (2013). Critical appraisal of ramucirumab (IMC-1121B) for cancer treatment: from benchside to clinical use. *Drugs*.

[8] Yi J. H., Lee J., Lee J. (2012). Randomised phase II trial of docetaxel and sunitinib in patients with metastatic gastric cancer who were previously treated with fluoropyrimidine and platinum. *British Journal of Cancer*.

[9] Lordick F., Kang Y. K., Chung H. C. (2013). Capecitabine and cisplatin with or without cetuximab for patients with previously untreated advanced gastric cancer (EXPAND): a randomised, open-label phase 3 trial. *The Lancet Oncology*.

[10] Ren Z., Sun J., Sun X., Hou H., Li K., Ge Q. (2017). Efficacy and safety of different molecular targeted agents based on chemotherapy for gastric cancer patients treatment: a network meta-analysis. *Oncotarget*.

[11] Ciliberto D., Staropoli N., Caglioti F. (2015). A systematic review and meta-analysis of randomized trials on the role of targeted therapy in the management of advanced gastric cancer: evidence does not translate?. *Cancer Biology & Therapy*.

[12] Wong H., Yau T. (2012). Targeted therapy in the management of advanced gastric cancer: are we making progress in the era of personalized medicine?. *The Oncologist*.

[13] Albarello L., Pecciarini L., Doglioni C. (2011). HER2 testing in gastric cancer. *Advances in Anatomic Pathology*.

[14] Nishida Y., Kuwata T., Nitta H. (2015). A novel gene-protein assay for evaluating HER2 status in gastric cancer: simultaneous analyses of HER2 protein overexpression and gene amplification reveal intratumoral heterogeneity. *Gastric Cancer*.

[15] Hamakawa T., Kukita Y., Kurokawa Y. (2015). Monitoring gastric cancer progression with circulating tumour DNA. *British Journal of Cancer*.

[16] Schwarzenbach H., Hoon D. S. B., Pantel K. (2011). Cell-free nucleic acids as biomarkers in cancer patients. *Nature Reviews Cancer*.

[17] Fang W. L., Lan Y. T., Huang K. H. (2016). Clinical significance of circulating plasma DNA in gastric cancer. *International Journal of Cancer*.

[18] Yang X., Zhuo M., Ye X. (2016). Quantification of mutant alleles in circulating tumor DNA can predict survival in lung cancer. *Oncotarget*.

[19] Campomenosi P., Gini E., Noonan D. M. (2016). A comparison between quantitative PCR and droplet digital PCR technologies for circulating microRNA quantification in human lung cancer. *BMC Biotechnology*.

[20] Whale A. S., Huggett J. F., Cowen S. (2012). Comparison of microfluidic digital PCR and conventional quantitative PCR for measuring copy number variation. *Nucleic Acids Research*.

[21] Hindson B. J., Ness K. D., Masquelier D. A. (2011). High-throughput droplet digital PCR system for absolute quantitation of DNA copy number. *Analytical Chemistry*.

[22] Kinugasa H., Nouso K., Tanaka T. (2015). Droplet digital PCR measurement of HER2 in patients with gastric cancer. *British Journal of Cancer*.

[23] Shoda K., Ichikawa D., Fujita Y. (2017). Monitoring the HER2 copy number status in circulating tumor DNA by droplet digital PCR in patients with gastric cancer. *Gastric Cancer*.

[24] Wong D., Moturi S., Angkachatchai V. (2013). Optimizing blood collection, transport and storage conditions for cell free DNA increases access to prenatal testing. *Clinical Biochemistry*.

[25] Eisenhauer E. A., Therasse P., Bogaerts J. (2009). New response evaluation criteria in solid tumours: revised RECIST guideline (version 1.1). *European Journal of Cancer*.

[26] Gevensleben H., Garcia-Murillas I., Graeser M. K. (2013). Noninvasive detection of HER2 amplification with plasma DNA digital PCR. *Clinical Cancer Research*.

[27] Dancey J. E., Bedard P. L., Onetto N., Hudson T. J. (2012). The genetic basis for cancer treatment decisions. *Cell*.

[28] Deng N., Goh L. K., Wang H. (2012). A comprehensive survey of genomic alterations in gastric cancer reveals systematic patterns of molecular exclusivity and co-occurrence among distinct therapeutic targets. *Gut*.

[29] Gomez-Martin C., Plaza J. C., Pazo-Cid R. (2013). Level of HER2 gene amplification predicts response and overall survival in HER2-positive advanced gastric cancer treated with trastuzumab. *Journal of Clinical Oncology*.

[30] Nakamura R., Yamamoto N., Onai Y., Watanabe Y., Kawana H., Miyazaki M. (2013). Importance of confirming HER2 overexpression of recurrence lesion in breast cancer patients. *Breast Cancer*.

[31] Cheng F., Su L., Qian C. (2016). Circulating tumor DNA: a promising biomarker in the liquid biopsy of cancer. *Oncotarget*.

